# Prognostic value of the postoperative neutrophil-lymphocyte ratio in solid tumors: A meta-analysis

**DOI:** 10.1371/journal.pone.0250091

**Published:** 2021-04-19

**Authors:** Meilong Wu, Shizhong Yang, Xiaobin Feng, Chengquan Li, Fei Yu, Jiahong Dong

**Affiliations:** 1 School of Clinical Medicine, Tsinghua University, Haidian District, Beijing, China; 2 Hepato-pancreato-biliary Center, Beijing Tsinghua Changgung Hospital, School of Clinical Medicine, Tsinghua University, Changping District, Beijing, China; 3 Institute for Precision Healthcare, Tsinghua University, Haidian District, Beijing, China; University Hospital Hamburg Eppendorf, GERMANY

## Abstract

**Purpose:**

Numerous studies have demonstrated that a variety of systemic inflammatory markers were associated with the survival of different tumors. However, the association between elevated postoperative neutrophil-lymphocyte ratio (postNLR) and long-term outcomes, including overall survival (OS), disease-free survival (DFS), in patients with solid tumors remains controversial. A systematic review was conducted to explore the association between the postNLR and long-term outcomes in solid tumors.

**Materials and methods:**

Relevant literature was identified using PubMed, Embase, Web of Science, and the Cochrane Library from the initiation of the databases to October 2020. Data were extracted from included studies reporting hazard ratio (HR) and 95% confidence intervals (CI), and were pooled using generic inverse-variance and random-effects modeling. 25 studies reporting on7539 patients were included in the analysis.

**Results:**

Elevated postNLR was associated with poor OS (HR 1.87, 95% CI = 1.53–2.28; *P* < 0.00001), and worse DFS (HR 1.69, 95% CI = 1.28–2.22; *P* = 0.0002). Subgroup analyses showed that the trend of the pooled HR for most of the subgroups was not changed, and the heterogeneity of the same tumor type was not obvious. However, there was no correlation between high postNLR obtained within 7days and poor DFS (n = 3, HR 1.25, 95CI% = 0.54–2.88; *P* = 0.60).

**Conclusions:**

Elevated postNLR might be a readily available and inexpensive biomarker for long-term outcomes in solid tumors. Multicenter and prospective studies are needed to explore the impact of the postNLR on the prognosis of solid tumors.

## Introduction

Cancer is becoming the leading cause of morbidity and mortality in every region of the world, and the global incidence of cancer is expected to increase from 12.7 million new cases in 2008 to 22.2 million by 2030 [1]. Surgery is critical to the treatment of cancer, and it is estimated that annually, 45 million surgical operations will be needed worldwide by 2030 [2]. Therefore, reliable and inexpensive biomarkers are needed to predict the survival of tumor patients and to distinguish subgroups of patients who will benefit from aggressive surgical treatment.

The mechanism of tumorigenesis is complex. The inflammatory response plays an important role in tumorigenesis, progression and metastasis [3,4]. Numerous studies have demonstrated that a variety of systemic inflammatory markers, such as preoperative neutrophil-lymphocyte ratio (NLR), were associated with the survival of different tumors [5,6]. As we all know, neutrophils promote tumorigenesis, progression and metastasis in multiple ways [7], while lymphocytes inhibit tumor growth, higher lymphocytes indicate a better prognosis for patients with cancer [8]. A high NLR indicates a decrease in the number of lymphocytes and an increase in the number of neutrophils. Changes in NLR may represent the balance of promoting or anti-tumor progression, and has prognostic value. Recently, a few studies have begun to focus on the impact of the postoperative NLR (postNLR) on tumor prognosis [9,10]. However, the prognostic value of the postNLR in solid tumors is controversial. A previous study showed that high postNLR was an independent prognostic factor for worse overall survival (OS) in tumor patients [9], but subsequent studies failed to demonstrate the prognostic value of the postNLR [10]. Therefore, the purpose of this meta-analysis is to investigate the association between the postNLR and long-term outcomes for solid tumors.

## Methods

The meta-analysis was conducted in accordance with the Preferred Reporting Items for Systematic Reviews and Meta-Analyses (PRISMA) [11].

### Search strategy

A systematic literature search of PubMed, EMBASE, Web of Science and the Cochrane Library was performed to select relevant articles from the initiation of the databases to October 09, 2019. No additional restrictions were applied to the searches. The following search strategies and keywords were used: (Neoplasm OR Neoplasia OR Tumor OR Cancer OR Carcinoma OR Malignancy) AND (Neutrophil OR lymphocyte) AND (postoperative).

### Selection criteria

Endnote X9 was used to screen duplicate documents. The literature was screened by title and abstract, and the full text was further reviewed to obtain qualified literature. Citation lists of the retrieved articles were screened manually to obtain relevant articles. The inclusion criteria were as follows: (1) patients with a pathologically confirmed diagnosis of cancer, and treated with surgery; (2) the postNLR cutoff was clearly defined; (3) postNLR was a categorical variable; (4) postNLR was included as a variable in outcome analysis. The exclusion criteria were as follows: (1) animal experiments; (2) review, letters, comments, and articles not related to the topic; (3) non-English articles.

### Quality assessment

Newcastle–Ottawa scale (NOS) were used to assess the quality of nonrandomized studies [12]. A score of 6–9 indicates that the quality of the article is high, while a score of 0–5 indicates that the quality of the article is low.

### Data extraction

The following data were extracted from the literature included in the study: first author, year of publication, recruitment period and region, tumor type, sample size, time of obtaining the postNLR, postNLR cutoff, hazard ratio (HR) and corresponding 95% confidence interval (CI) for OS and disease-free survival (DFS). The HR was preferentially extracted from multivariate analysis, otherwise from univariate analysis, or obtained from Kaplan–Meier curves using Engauge Digitizer 4.1 [13].

### Data analysis

RevMan 5.3 analysis software was used to conduct the meta-analysis. The estimates for HR were pooled and weighted by generic inverse variance and then computed by random-effects modeling. Odds ratio (OR) was synthesized to explore the relationship between high postNLR and clinicopathological indicators. Heterogeneity was assessed using *I*^*2*^ statistics. If *I*^*2*^ > 50%, the heterogeneity was considered statistically significant [14]. Publication bias was assessed by visually inspecting funnel plots. All statistical tests were two-sided, and statistical significance was defined as *P* < 0.05.

## Results

### Study selection and patient characteristics

**Fig 1:**
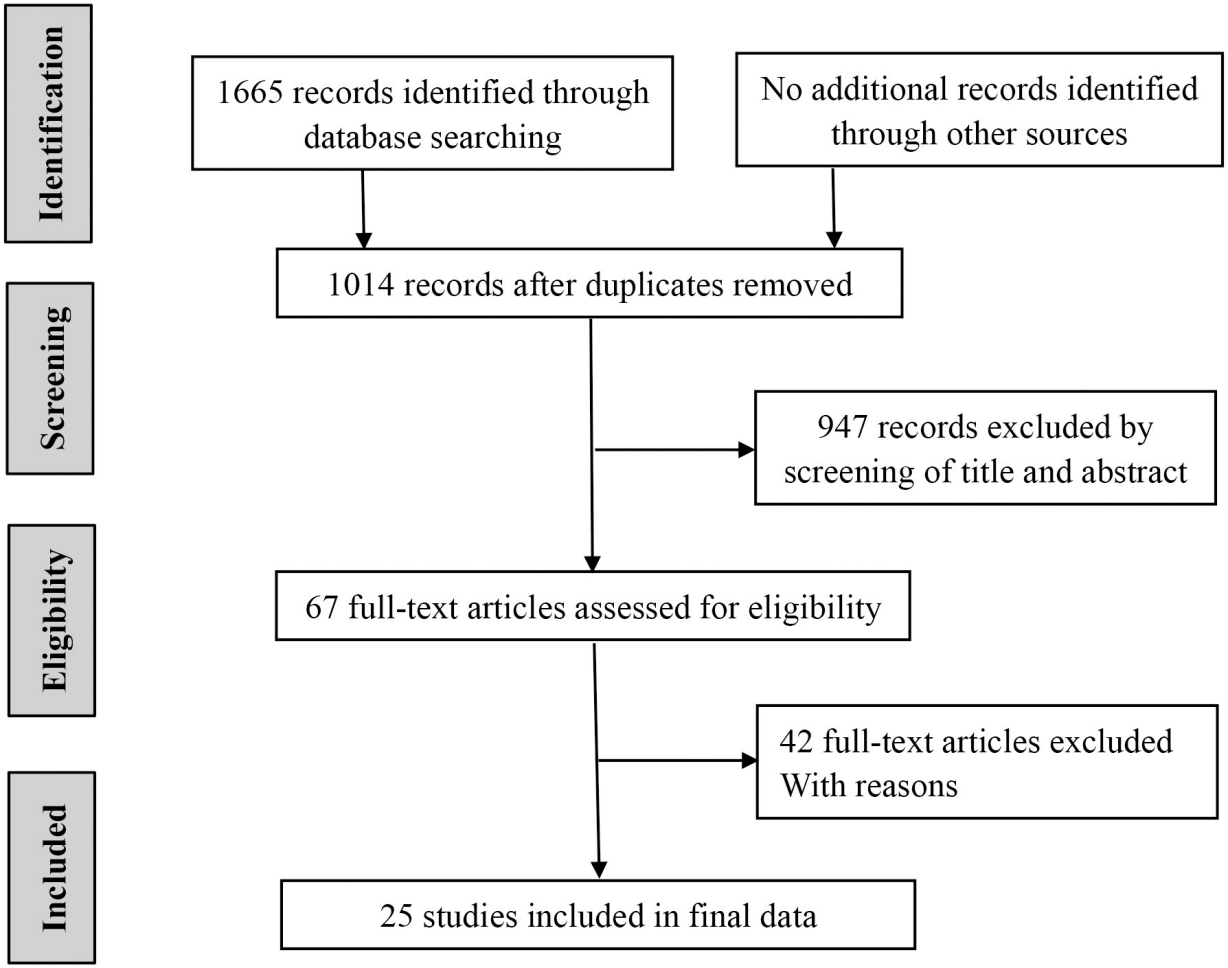
Selection of studies included in the analysis.

Fig 1 shows the detailed steps of the literature search. A total of 1665 articles were retrieved. No additional records were identified through other sources. 651 duplicate records were excluded. Then, 947 references were eliminated by screening the titles and abstracts. Of the remaining 67 potentially relevant studies, 42 studies were excluded because they fulfilled one of the exclusion criteria. Finally, 25 studies (all retrospective) reporting on 7539 patients were eligible to be included in the present meta-analysis [9,10,15–37]. As shown in Table 1, the studies were conducted in Belgium (1 study), Japan (8 studies), Switzerland (1study), China (7 studies), Poland (1 study), Korea (5 studies), USA (2 studies). The 25 studies were published between 2012 and 2020. 19 of them studied OS, 15 discussed DFS. The surgery in 11 studies was radical surgical treatment, two of which also performed chemoradiation or chemotherapy. The remaining 14 studies underwent resection, and 5 of them received chemotherapy or radiotherapy for some patients. The median of NLR was 3.28 (1.8–14.10). The median of sample size was 176 (93–2302). The NOS score ranged from 6 to 8 points, indicating that the quality of the literature included in the study is high.

**Table 1:** Baseline characteristics of the included studies.

studies	tumor type	treatment	region	sample	Inclusion period	NLR/time obtained	outcomes	NOS
Albisinni 2019 [15]	bladder cancer	CR	Belgium	134	2013–2018	5.58/ 1 month	OS, DFS	8
Bojaxhiu 2018 [16]	head and neck cancer	R, RC	Switzerland	186	2007–2010	3.28/ <10 days	OS, DFS	6
Cui 2020 [17]	colorectal cancer	R	China	146	2011–2014	2.96/≥7 days	DFS	7
Guo 2018 [18]	colorectal cancer	R	China	135	2009–2014	3.64/>1 month	OS, DFS	7
Hayama 2020 [19]	colorectal cancer	CR	Japan	176	2012–2016	3.1/ 7th day	DFS	6
He 2017 [20]	synchronous colorectal cancer	CR	China	114	2009–2013	10.5/≤3 days	OS, DFS	6
Hoshimoto 2020 [21]	pancreatic cancer	R	Japan	211	1995–2016	1.8/ 18–86 days	OS	7
Jakubowska 2020 [22]	colorectal cancer	R	Poland	144	2014–2016	3.3/ 3 days	DFS	6
Jang 2017 [23]	prostate cancer	CR	Korea	2302	2000–2010	3.5/ 2–3 months	OS	7
Jin 2016 [24]	non-small cell lung cancer	R	China	123	2007–2010	3.9/ >1 month	OS, DFS	8
Kang 2016 [9]	bladder cancer	CR	Korea	385	1999–2012	2/ 3–4 month	OS	7
Kim 2019 [25]	pancreas adenocarcinoma	CRC	Korea	178	2010–2018	2.535/ 1 months	OS, DFS	6
Kim 2012 [26]	stomach cancer	R	Korea	93	2004–2009	7.7/ third day	DFS	8
Li 2018 [27]	colon cancer	CR	China	344	2012–2015	3/ 1–3 months	OS	6
Lin 2019 [28]	oropharyngeal cancer	R/RR	USA	108	1997–2017	6.2/ 3 months	OS, DFS	6
Lin 2018 [29]	palatine tonsil cancer	RR	USA	99	1997–2013	11.875/<2.5 months	OS	6
Miyatani 2018 [30]	gastric cancer	CR	Japan	280	2001–2013	1.8/ 1 month	OS	6
Nishihara 2019 [31]	upper tract urothelial carcinoma	CR	Japan	134	2004–2015	2.5/ 1–2 month	OS	8
Ohno 2012 [32]	clear cell renal cell carcinoma	R	Japan	250	1990–2008	2.7/ 3 months	DFS	6
Paik 2014 [33]	colorectal cancer	R	Korea	600	2006–2009	5/ NA	OS	7
Pu 2019 [34]	pancreatic ductal adenocarcinoma	R	China	97	2012–2016	14.1/ NA	OS, DFS	7
Shibutani 2015 [35]	colorectal cancer	CR	Japan	254	2006–2011	3/ 29 (23–36) days	OS	7
Tanaka 2018 [36]	gastric cancer	RC	Japan	170	2006–2015	1.99/ NA	DFS	6
Zhou 2016 [10]	gastric cancer	CR/CRC	China	360	2006–2008	6.19/≥5 days	OS	8
Zhou 2018 [37]	colorectal cancer	R	China	516	2007–2015	2.41/ 1 month	OS, DFS	7

CR, curative resection; CRC, curative resection and chemotherapy; R, resection; RR, resection and radiotherapy; RC, resection and chemotherapy; NLR, neutrophil-lymphocyte ratio; OS, overall survival; DFS, disease-free survival; NOS, Newcastle–Ottawa scale; NA, not available.

### PostNLR and OS

**Fig 2:**
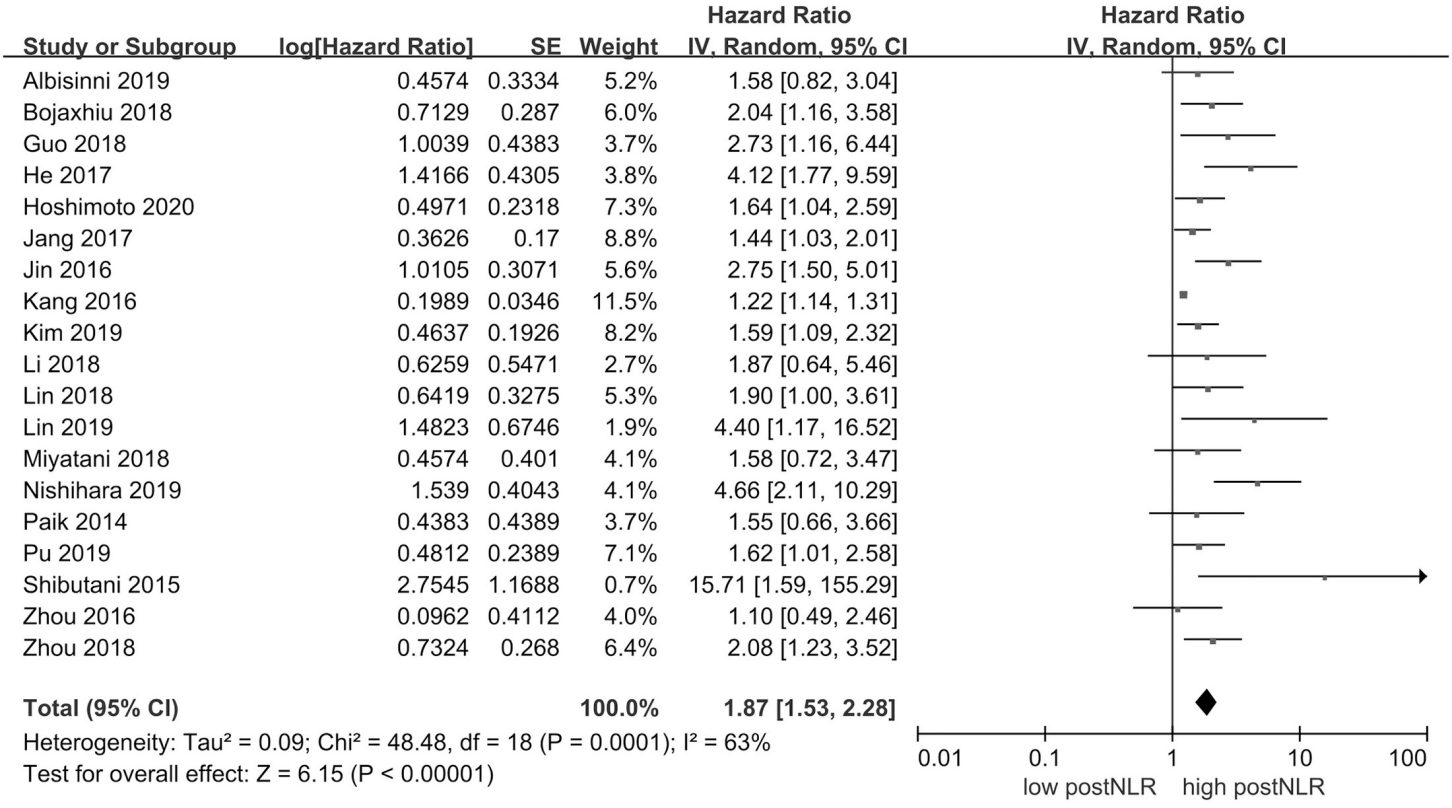
Results of the meta-analysis on pooled hazard ratio values for overall survival.

19 studies comprising a total of 6560 patients reported HR for OS. Fig 2 shows that high postNLR was associated with poor OS (HR 1.87, 95% CI = 1.53–2.28, *P* < 0.00001). There was statistically significant heterogeneity (*I*^*2*^ = 63%, *P* = 0.0001).

### PostNLR and DFS

**Fig 3:**
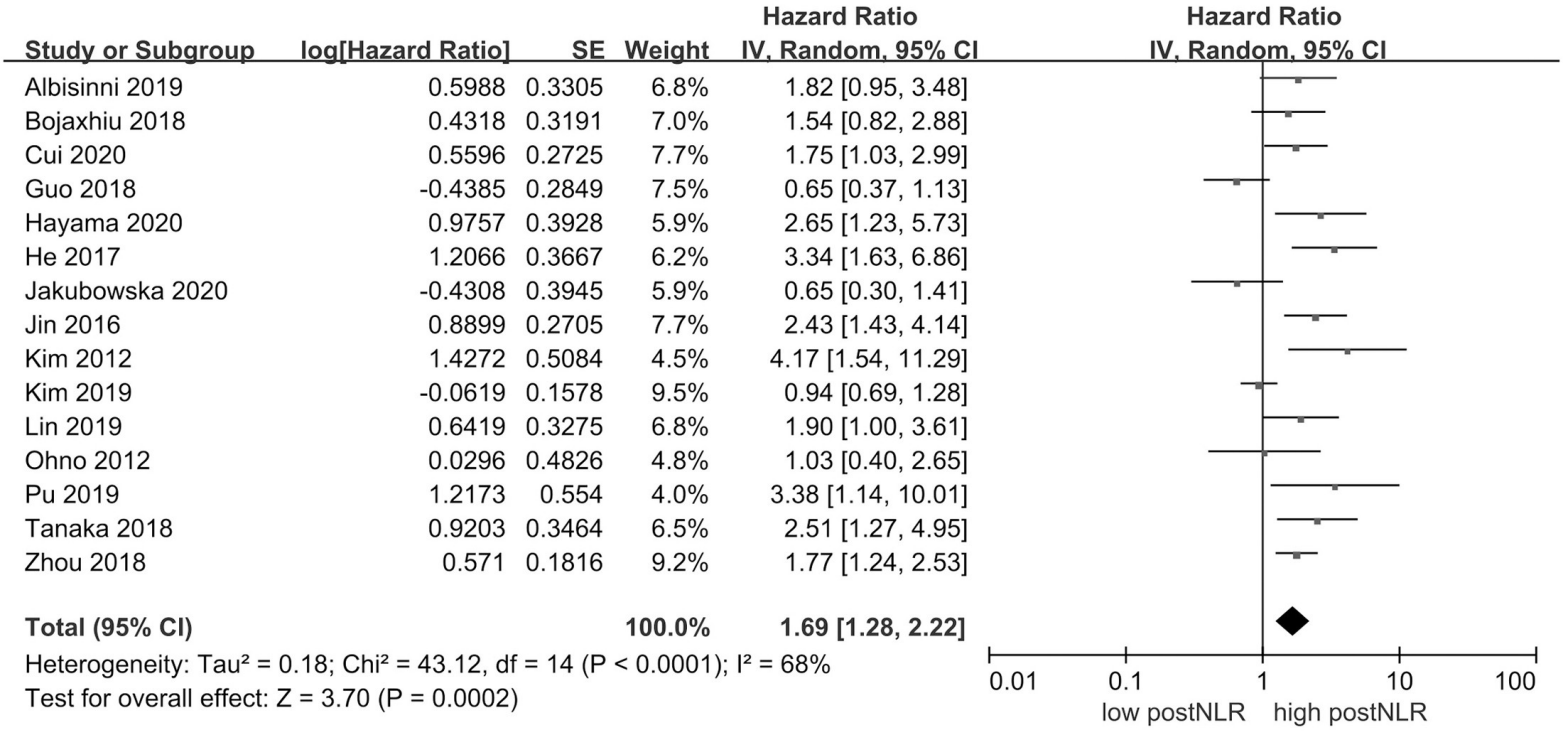
Results of the meta-analysis on pooled hazard ratio values for disease-free survival.

15 studies with 2570 individuals were included in the analysis of postNLR and DFS. Fig 3 demonstrated that high postNLR was associated with worse DFS (HR 1.69, 95% CI = 1.28–2.22, *P* = 0.0002). There was evidence of significant heterogeneity across the included studies (*I*^*2*^ = 68%, *P* < 0.0001).

### Subgroup analysis

A subgroup analysis of the tumor type, treatment and time of obtaining postNLR was used to investigate the correlation between high postNLR and OS or DFS. Subgroup analyses showed that the trend of the pooled HR for most of the subgroups was not changed (Table 2). Stratified analysis by tumor type showed that studies conducted in colorectal cancer (n = 4, HR 2.25, 95% CI = 1.40–3.61, *P* = 0.008), bladder cancer (n = 2, HR 1.22, 95% CI = 1.14–1.31, *P*<0.00001), and pancreatic cancer (n = 3, HR 1.61, 95C%CI = 1.26–2.07, *P* = 0.0001) were significantly associated with reduced OS. A similar result indicated that high post NLR was associated with worse DFS of colorectal cancer. Subgroup analysis revealed that the heterogeneity of the same tumor type was small. However, there was no significant difference in gastric cancer between high postNLR and poor OS (n = 2, HR 1.32,95%CI = 0.75–2.33, *P* = 0.33), and in colorectal cancer between high postNLR and worse DFS (n = 5, HR 1.30, 95%CI = 0.78–2.16, *P* = 0.32).

**Table 2:** Subgroup analysis of the association between high postNLR and the long-term outcomes of solid tumors.

	Overall survival					Disease-free survival				
subgroup	studies	patients	HR (95CI%)	*I*^*2*^	*P*	studies	patients	HR 95CI%	*I*^*2*^	*P*
Tumor type										
Colorectal cancer	4	1505	2.25 (1.40–3.61)	20%	0.0008	5	1117	1.30 (0.78–2.16)	75%	0.32
Gastric cancer	2	640	1.32 (0.75–2.33)	0%	0.33	2	263	2.95 (1.68–5.17)	0%	0.0002
Bladder cancer	2	519	1.22 (1.14–1.31)	0%	<0.00001	-	-	-	-	-
Pancreatic cancer	3	486	1.61 (1.26–2.07)	0%	0.0001	-	-	-	-	-
Treatment										
Curative resection	8	3947	1.95 (1.35–2.81)	73%	0.0004	3	424	2.46 (1.64–3.70)	0%	<0.0001
resection	6	1682	1.91 (1.52–2.41)	0%	<0.00001	8	1504	1.55 (1.01–2.37)	71%	0.04
NLR time										
<7 days	-	-	-	-	-	3	351	1.25 (0.54–2.88)	83%	0.60
≥7 days	13	5104	1.83 (1.45–2.30)	65%	<0.00001	9	1766	1.50 (1.09–2.05)	68%	0.01

NLR, neutrophil-lymphocyte ratio; HR, hazard ratio; CI, confidence interval.

As shown in Table 1, 8 studies of curative resection studied OS and 3 researches studied DFS. 6 of the 14 resection studies studied OS, and 8 studies studied DFS. The included studies are divided into 2 subgroups based on treatment methods (Table 2). Subgroup analysis showed that the high postNLR group had adverse effects on OS (curative resection: n = 8, HR 1.95, 95%CI = 1.35–2.81, *P* = 0.0004; resection: n = 6, HR 1.91, 95CI% = 1.52–2.41, *P*<0.00001) and DFS (curative resection: n = 3, HR 2.46, 95%CI = 1.64–3.70, *P*<0.0001; resection: n = 8, HR 1.55, 95CI% = 1.01–2.37, *P* = 0.04) compared to the low postNLR group.

Stratified by the time to obtain postNLR showed that high postNLR in the group greater than 7 days has adverse effects on OS and DFS (Table 2). However, there was no correlation between high postNLR obtained within 7days and poor DFS (n = 3, HR 1.25, 95CI% = 0.54–2.88, *P* = 0.60).

### Correlation between postNLR and Clinicopathological indicators

As shown in Table 3, five studies reported the relationship between age (≥60 years and <60years) and high postNLR. There was no correlation between high postNLR and age (OR 1.04, 95CI% = 0.65–1.67, *P* = 0.87). Similar results showed that there was no significant correlation between gender and high postNLR (*P* = 0.16).

**Table 3:** Correlation between high postNLR and clinicopathological indicators.

Clinicopathological indicators	studies	patients	OR (95CI%)	*I*^*2*^	*P*
Age≥60 years VS Age<60 years	5	840	1.04 (0.65–1.67)	59%	0.87
Male VS female	10	1672	0.78 (0.56–1.10)	59%	0.16
CEA normal VS elevated	4	576	0.67 (0.41–1.09)	28%	0.11
AJCC (7th) III+IV VS I+II	2	281	7.67 (0.08–709.58)	94%	0.38
AJCC (7th) T III+IV VS T I+II	3	601	0.29 (0.06–1.31)	91%	0.11

NLR, neutrophil-lymphocyte ratio; OR, odds ratio; CEA, carcinoembryonic antigen; AJCC (7th), 7th edition of the American Joint Committee on Cancer staging system.

Four studies were pooled to explore the relationship between high postNLR and tumor marker (carcinoembryonic antigen, CEA) (Table 3). The result suggested that there was no significant difference between elevated CEA and high postNLR (OR 0.67, 95CI% = 0.41–1.09, *P* = 0.1).

The clinical staging systems and T stage were synthesized to explore the correlation between tumor stage and high postNLR (Table 3). Different clinical staging systems were used in the original research, which limited the number of studies that could quantitatively compare the relationship between postNLR and clinical staging systems. Finally, 4 studies [17,18,20,27] of the 7th edition of the American Joint Committee on Cancer (AJCC 7th) staging system were included in quantitative synthesis to explore the relationship between AJCC (7th) staging system and high postNLR. The results indicated that there was no significant difference between high postNLR and AJCC (7th) staging system (OR 7.67, 95CI% = 0.08–709.58, *P* = 0.38), and T stage (OR 0.29, 95CI% = 0.06–1.31, *P* = 0.11).

### Publication bias

**Fig 4:**
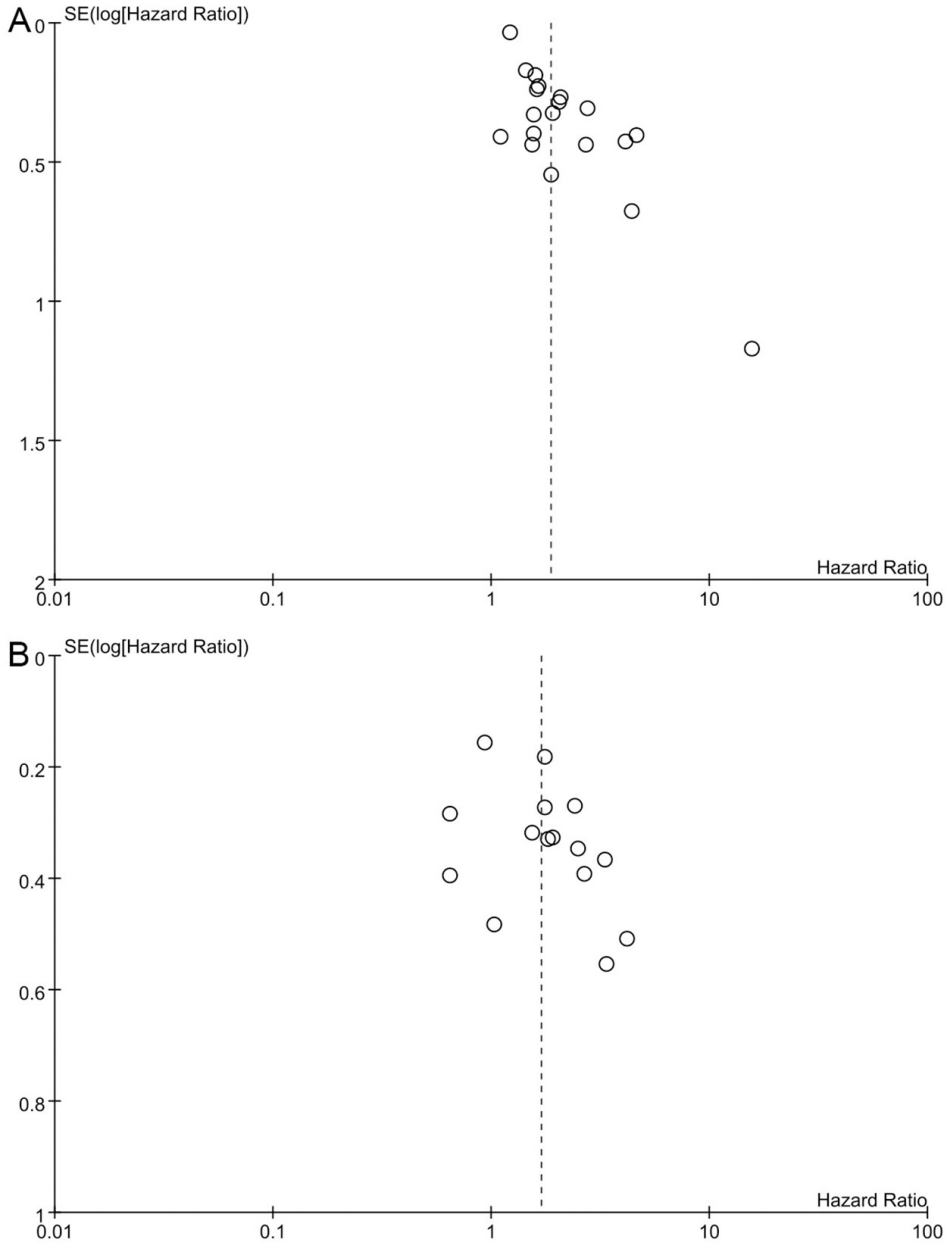
Funnel plot of the hazard ratio for (A) overall survival; (B) disease-free survival.

As shown in Fig 4, the included studies were not symmetrically distributed, indicating that there was evidence of publication bias for OS, and DFS.

## Discussion

To the best of our knowledge, this is the first meta-analysis investigating the association between the postNLR and long-term outcomes (OS and DFS) in solid tumors. The current systematic review and meta-analysis of 25 studies including 7539 patients provided solid evidence of an association between a high postNLR and reduced long-term outcomes. This result was further confirmed by subgroup analysis. Subgroup analysis of different types of solid tumors showed that high postNLR was associated with poor long-term outcomes, and the heterogeneities within the subgroup were not obvious. Further analysis found that there was no correlation between clinicopathological indicators including tumor stage and postNLR. Our previous study found that the preoperative inflammatory indicators were related to tumor burden such as tumor size, while the preoperative tumor burden was not correlated with postoperative inflammatory indicators [38]. The possible reason was that after the primary tumor burden was removed, the host’s immune response has changed [39]. This meta-analysis further confirmed that there was no significant correlation between postNLR and preoperative tumor burden such as tumor size and tumor stage. The results from the present study demonstrated that the postNLR could serve as a readily available and inexpensive biomarker to predict the long-term outcomes of patients with solid tumors after surgery. It is worth noting that this study found that there was no significant difference between postNLR obtained within 7 days after surgery and poor DFS. The possible reason is that surgical stress and wound healing have an impact on inflammatory indicators [40]. Therefore, the prognostic value of postNLR in the early postoperative period may be limited.

The mechanism for the correlation between increased postNLR and worse survival outcomes in patients with solid tumors is complex. Recent studies have demonstrated that the NLR represents the balance between antitumor immune function and the inflammatory response [41]. Increased preoperative NLR was associated with lymph node metastasis and distant metastasis [42] as well as treatment resistance [43]. We suspect that the postNLR may play an important role in the activation of micrometastases and resistance to treatment. A high NLR indicates a decrease in the number of lymphocytes and an increase in the number of neutrophils. Generally, neutrophils play a critical role in tumorigenesis, progression and metastasis in multiple ways, including both direct effects on cancer cells and indirect effects on the tumor microenvironment [7]. Tumor growth can be directly enhanced by neutrophil elastase [44]. Cools et al [45] reported that tumor cell trapping within neutrophil extracellular traps was associated with increased micrometastases. As reported, a high NLR correlates with increased interleukin (IL)-6, IL-8 and regulatory T cell expression [46]. IL-6 increases vascular endothelial growth factor release, stimulates defective angiogenesis [47], and enhances chemoresistance, resistance to apoptosis and invasion [48]. Comparatively, the importance of lymphocytes has been highlighted in systematic review in which high tumor-infiltrating lymphocyte densities were associated with improved survival outcomes in cancer [8]. Moreover, the apoptosis of tumor-infiltrating lymphocytes mediates resistance to cancer immunotherapy [49]. One study showed that an early decline in the NLR at 6 weeks in patients with metastatic renal cell carcinoma receiving immune checkpoint blockade was associated with improved survival outcomes [50]. Therefore, an elevated NLR marked the suppression of host immunity. Our findings support this result that high postNLR indicates poor prognosis for solid tumors.

Our study has some strengths that need to be addressed. First, to the best of our knowledge, this is the first meta-analysis to explore the association between the postNLR and long-term outcomes in solid tumor patients. Second, heterogeneities in most of the subgroup analyses for OS were not obvious. Third, we developed a reproducible and systematic search strategy for the major medical databases, and the references of the initially selected studies were screened for additional studies that were not included in the database search. Finally, this study suggests that during the entire treatment process, we should not only focus on the preoperative immune or inflammatory status but also on the impact of the postoperative inflammatory status on tumor progression and treatment resistance.

There are several limitations in our study. First, all studies are retrospective with immeasurable deviations. Second, the limited number of studies for subgroup analysis may affect the reliability of the results. Third, HR and corresponding 95% CI of some included studies were extracted by Kaplan–Meier curves, affecting the accuracy of the results. Furthermore, we found evidence of publication bias, as shown in Fig 4. Unpublished gray literature, mainly with negative results, may have affected the pooled estimates. Forth, the NLR of different tumors may vary. NLR values may be affected by tumor type, and especially postoperative detection time because surgical stress and wound healing have an impact on inflammatory indicators. At the same time, most studies come from East Asia, and there may be ethnic differences that restrict the generalization and reliability of the results. Fifth, confounding factors such as neoadjuvant therapy may have a potentially unclear influence on this result. In addition, 14 of the 25 studies did not state in the original text whether it was a radical resection, which may affect the oncologic outcome itself. Finally, due to the limitation of the number of studies and the different tumor staging systems, we were unable to explore the relationship between AJCC (7th) I-IV, T I-IV and high postNLR, which may have unknown effects.

## Conclusion

Elevated postNLR might be a promising biomarker for OS and DFS in solid tumors. Routine monitoring of postNLR during postoperative follow-up may be helpful in predicting the prognosis of patients with tumors Multicenter and prospective studies are needed to explore the impact of the postNLR on the prognosis of solid tumors.

## Supporting information

S1 Checklist PRISMA checklist. (DOCX)

S1 Dataset Dataset. (ZIP)
