# Expression of Concern: Prognostic value of the postoperative neutrophil-lymphocyte ratio in solid tumors: A meta-analysis

**DOI:** 10.1371/journal.pone.0282192

**Published:** 2023-02-16

**Authors:** The PLOS ONE Editors

After this article was published, similarities were noted between this article [1] and submissions by other research groups which call into question the validity and provenance of the reported results.

In response to queries about these concerns, the first author provided the underlying data in S1–S3 Files.

The authors commented on aspects of how data were collected and analyzed for this study, but overall their responses did not fully resolve the concerns.

In light of these issues, the *PLOS ONE* Editors issue this Expression of Concern.

## Supporting information

S1 File The underlying data used for the analysis in this article [1] in Excel format. (XLSX)

S2 File The underlying data used for the analysis in this article [1] in rm5 format. (RM5)

S3 File Underlying data for the data retrieval, extraction process and results used for the analysis in this article [1]. (RAR)
